# Analysis of maxillary asymmetry before and after treatment of functional posterior cross-bite: a retrospective study using 3D imaging system and deviation analysis

**DOI:** 10.1186/s40510-023-00494-z

**Published:** 2023-12-11

**Authors:** Vincenzo Ronsivalle, Gaetano Isola, Gianmarco Lo Re, Mattia Boato, Rosalia Leonardi, Antonino Lo Giudice

**Affiliations:** Department of General Surgery and Medical-Surgical Specialties, Section of Orthodontics, University of Catania, Policlinico Universitario “Gaspare Rodolico - San Marco”, Via Santa Sofia 78, 95123 Catania, Italy

**Keywords:** Digital orthodontics, Asymmetry, Maxillary expansion, Functional posterior cross-bite

## Abstract

**Background:**

Previous evidence would suggest that subjects affected by functional posterior cross-bite (FPXB) present an asymmetric morphology of the maxilla. However, no evidence is available concerning the morphology (symmetry/asymmetry) of the maxilla after treatment of FPXB. This study aimed to investigate the volumetric and morphological changes of the palate in FPXB subjects treated with maxillary expansion and to compare these data with an untreated control group. The study sample included 20 FPXB subjects (mean age 8.1 ± 0.9 years) who underwent maxillary expansion (MEG group) and 21 FPXB subjects (mean age 7.7 ± 1.2 years) as controls (CG group). Digital models were recorded at T0 (first observation) and T1 (12–18 months after first observation) and analyzed to assess palatal volume and symmetry. Deviation analysis and percentage matching calculation were also performed between original and mirrored palatal models for each patient. All data were statistically analyzed for intra-timing, inter-timing and inter-groups assessments.

**Results:**

At T0, the cross-bite side (CBS) was significantly smaller than non-cross-bite side (non-CBS) in both groups (*p* < 0.05). At T1, the CBS/non-CBS difference reduced significantly in the MEG group (*p* < 0.05) while slightly worsened in the CG, however without statistical significance (*p* > 0.05). The matching percentage of the palatal models improved significantly at T1 in the MEG group (T0 = 74.02% ± 9.8; T1 = 89.95% ± 7.12) (*p* < 0.05) while no significant differences were recorded in the CG (T0 = 76.36 ± 8.64; 72.18% ± 9.65) (*p* > 0.05).

**Limitations:**

The small sample size and the retrospective design of the study represent two limitations that should be overcome with further clinical trials.

**Conclusions:**

Subjects with FPXB present an asymmetric development of the maxillary vault that improves after reestablishment of normal occlusion following maxillary expansion.

**Supplementary Information:**

The online version contains supplementary material available at 10.1186/s40510-023-00494-z.

## Background

Posterior cross-bite represents a malocclusion with a documented prevalence between 7 and 23% in mixed and deciduous dentition [1, 2]. It can occur bilaterally or unilaterally and generally indicates the presence of transverse maxillary hypoplasia [3]. Unilateral posterior cross-bite is often associated with a mild bilateral maxillary constriction which causes occlusal interferences in centric relation, provoking mandibular shift toward the cross-bite in centric occlusion [4]. This condition is recognized as functional posterior cross-bite (FPXB). Skeletal maxillary expansion or dento-alveolar expansion are the primary treatment options to correct FPXB in children [5]. The early treatment of FPXB is encouraged to restore maxillary transverse dimension, increase the skeletal effectiveness and reduce any side effects of later treatment [6] while it is also considered effective to restore mandibular posture and prevent the subsequent asymmetric pattern of mandibular growth [5]. Also, early treatment of FPXB has been advocated to eliminate or prevent compensatory skeletal and dento-alveolar adaptation that could occur to maintain a stable function and occlusion in presence of mandibular asymmetry [4].

In this regard, recent evidence would suggest that FPXB can be associated with asymmetric pattern of development of the maxillary arch [4]. Using digital surface analysis, the authors founded that FPXB in mixed dentition could be associated with a symmetric contraction of the basal bone and asymmetry of the alveolar processes, with the cross-bite side being narrower than non-cross-bite side.

As second level of clinical evidence, it would be helpful to determine whether the correction of the cross-bite could positively influence the residual development of the maxilla in growing individuals, or if the existing asymmetry should be regarded as stable. However, there are not studies in literature dealing with the assessment of maxillary symmetry/asymmetry after correction of FPXB, except for one study that investigated the craniofacial asymmetry in children before and after rapid maxillary expansion (RME) [7]. The authors found that maxillary asymmetry improves after maxillary expansion; however, further studies are required to corroborate or contrast these findings [7].

Nowadays, with progresses in scanning systems and reverse engineering software for medical applications, 3D anatomical model can be mirrored and then superimposed to evaluate growth/treatment changes through the Euclidean distance measurements or root mean square (RMS) value [4, 8, 9]. Also, quantitative data can be visualized on a 3D color-map providing qualitative assessment of the morphological changes in distinct colors using a technique called ‘surface-to-surface’ analysis.

In this regard, the aim of the present study was to investigate the volumetric and morphological changes of the palate in subjects affected by FPXB treated with maxillary expansion, and to compare these data with an untreated control group, using a specific 3D imaging technology for the analysis of the asymmetry. The present study could provide additional diagnostic insights in the clinical orthodontic management of maxillary asymmetry in growing subjects. The null hypothesis was the absence of significant differences between the baseline data of palate symmetry/asymmetry and the same data recorded after maxillary expansion.

## Methods

### Study design

The present retrospective study received the approval of the Institutional Ethical Committee of the University of Catania (protocol n. 154/2022/PO) and has been carried out following the Helsinki Declaration on medical protocols and ethics. Before starting the orthodontic treatment, signed informed consent was obtained from parents.

### Settings

The study sample consisted of 41 patients with diagnosis of transverse maxillary deficiency (mean age 7.8 ± 1.1 years) who underwent orthodontic evaluation and treatment with maxillary expansion at the Department of Orthodontics of the University of Catania between September 2014 and December 2022.

Subjects in the treated group (MEG group = 20 subjects, mean age 8.1 ± 0.9 years) received a Hyrax maxillary expander anchored on the second deciduous molars with extended arms alongside the enamel-gingival junction of posterior teeth up to upper deciduous canines (Additional file 1: Fig. S1). The expansion protocol involved three activations per week (alternate days) and was interrupted once overexpansion was achieved, i.e., when the mesio-palatal cusps of the maxillary first molars were in contact with the buccal cusps of the mandibular first molars. The appliance was kept in place for 6 months as retention and during this period patients did not receive other orthodontic appliances. Dental impressions with bite registrations were taken before treatment (T0) and after 12 to 18 months (T1) without the appliance in place. In the control group (CG group = 21 subjects, 7.7 ± 1.2 years), dental impressions were taken at the first consultation (T0) and after 12 to 24 months as part of new diagnostic records. Plaster models at T0 and T1 were digitalized using D2000 3D desktop scanner (3Shape, Copenhagen, Denmark).

### Participants

The study sample was retrieved according to the following criteria: inclusion criteria = unilateral posterior cross-bite of at least two posterior teeth, mandibular shift toward the cross-bite site ≥ 2 mm in centric occlusion and not in centric relation (FPXB), class I or edge-to-edge molar relationship; exclusion criteria = anterior cross-bite, mobility of posterior deciduous teeth, previous orthodontic treatment, asymmetric design of expander or additional components in the expander framework (resin, tongue grid), permanent dentition, skeletal maturation (CVMS) ≥ Stage 3, cranial deformities or syndromic conditions. The MEG group included 20 participants who received skeletal maxillary expansion after the orthodontic consultation, instead the CG group included 21 subjects matching the same inclusion/exclusion criteria who delayed the orthodontic treatment one year after receiving an orthodontic consultation. Reasons for treatment delay were not related to specific orthodontic clinical conditions but rather influenced by subjective factors such as financial constraints or parental skepticism toward early intervention, as well as social and health restrictions during the COVID-19 pandemic. Additional file 2: Fig. S2 showed the flow chart with detailed description of the retrospective sample selection.

In the present study, the Strengthening the Reporting of Observational Studies (case–control studies) in Epidemiology (STROBE) were used as a reporting template (Additional file 3: Table S1) [10].

### Measurements

**Fig. 1:**
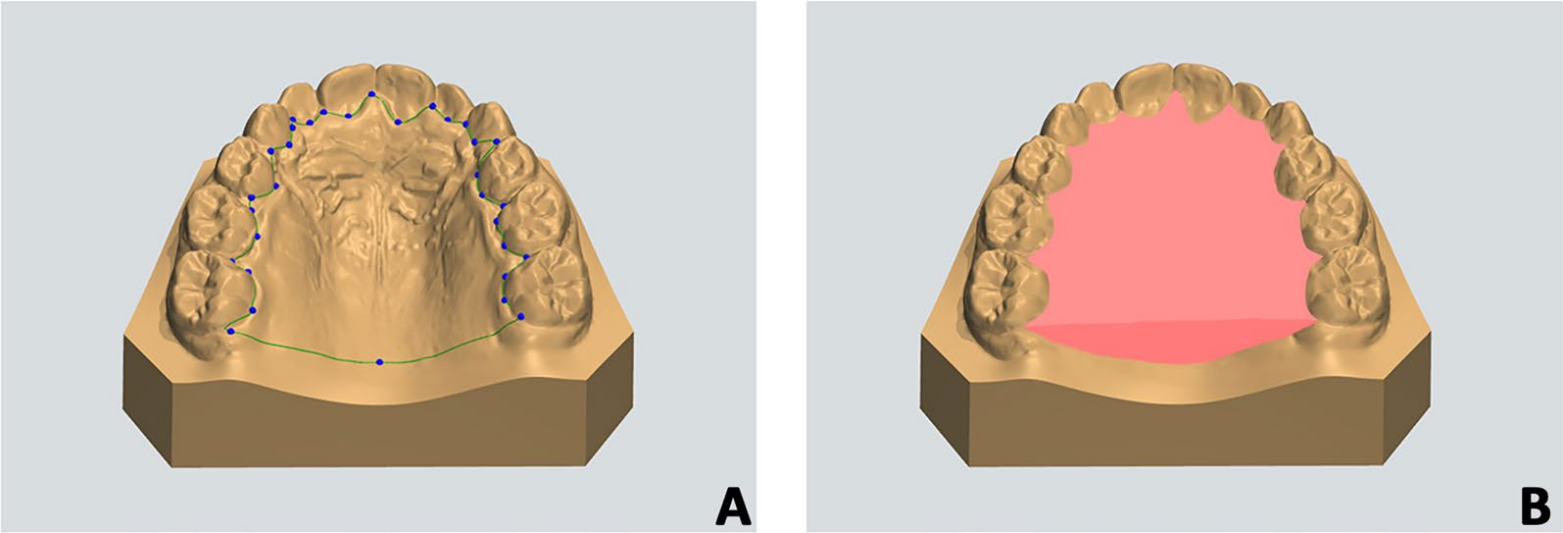
Segmentation of the palate and generation of maxillary reference model. **A**, **B** The anatomy of the palatal was isolated by generating a gingival plane passing through all the most apical points of the dento-gingival junction of all teeth, from the right 1st molar to the left 1st molar

All maxillary digital models were imported into Ortho Analyzer software (3Shape A/S, Copenhagen, Denmark) to perform the segmentation of the palate at T0 and T1. In particular, the anatomy of the palate was isolated by generating a gingival plane passing through all the most apical points of the dento-gingival junction of all teeth, from the right 1st molar to the left 1st molar (Fig. 1A, B).

**Fig. 2:**
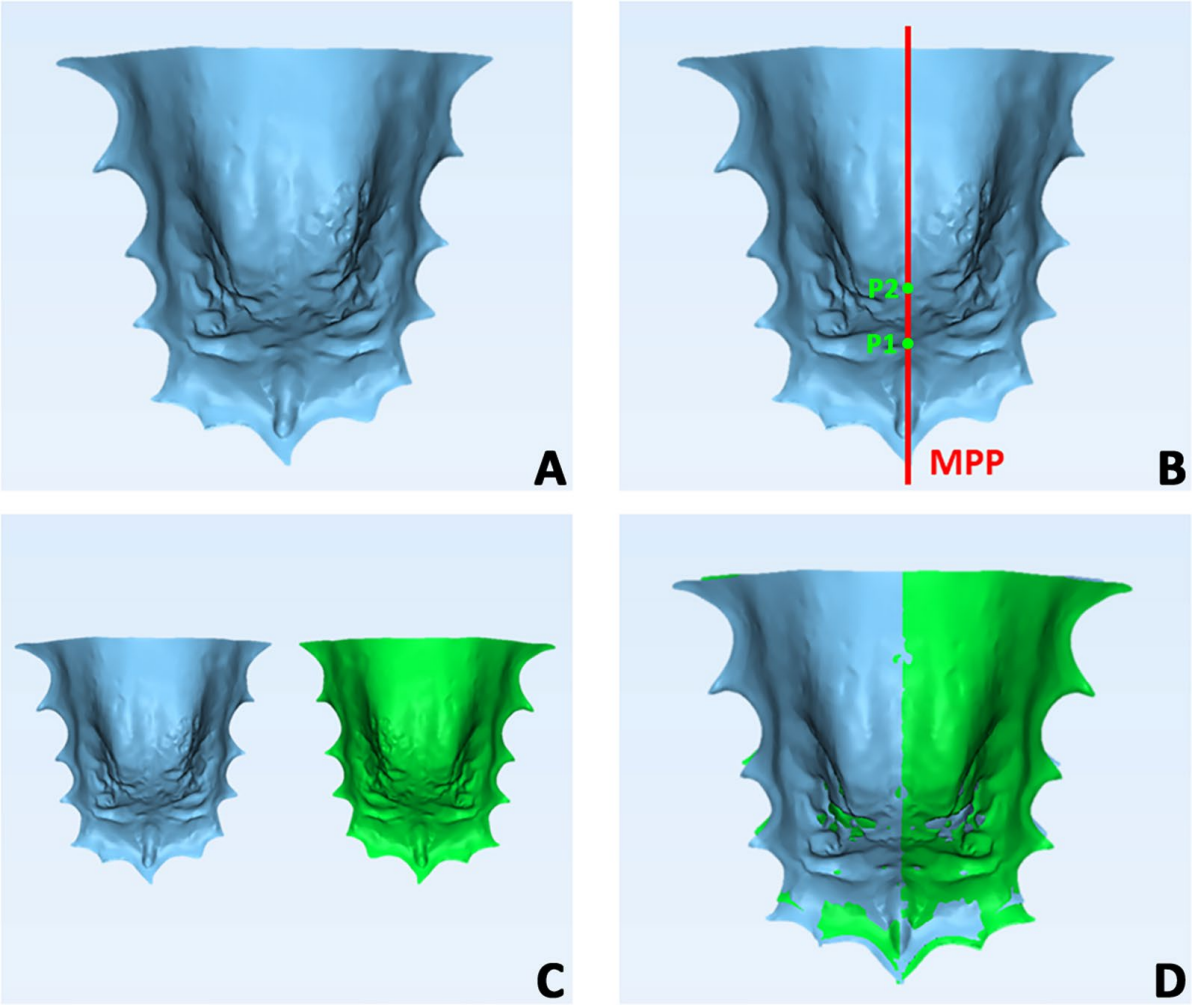
Mirroring process of the palate and superimposition between original (reference) and mirrored model. **A**, **B** The median palatal plane (MPP) was drawn through two landmarks detected along the median palatal raphe and showed in red. The first landmark identified the point on the median palatal raphe adjacent to the second ruga (Point 1). The second landmark was placed on the median palatal raphe 1 cm distal to the first point (Point 2). **C** Generation of duplicated mirrored model of the palate (blue model = original, green model = mirrored). **D** Superimposition of the original and mirrored models using the MPP plane and its perpendicular plane as reference and final superimposition adjustment using ‘best-fit’ alignment algorithm

**Fig. 3:**
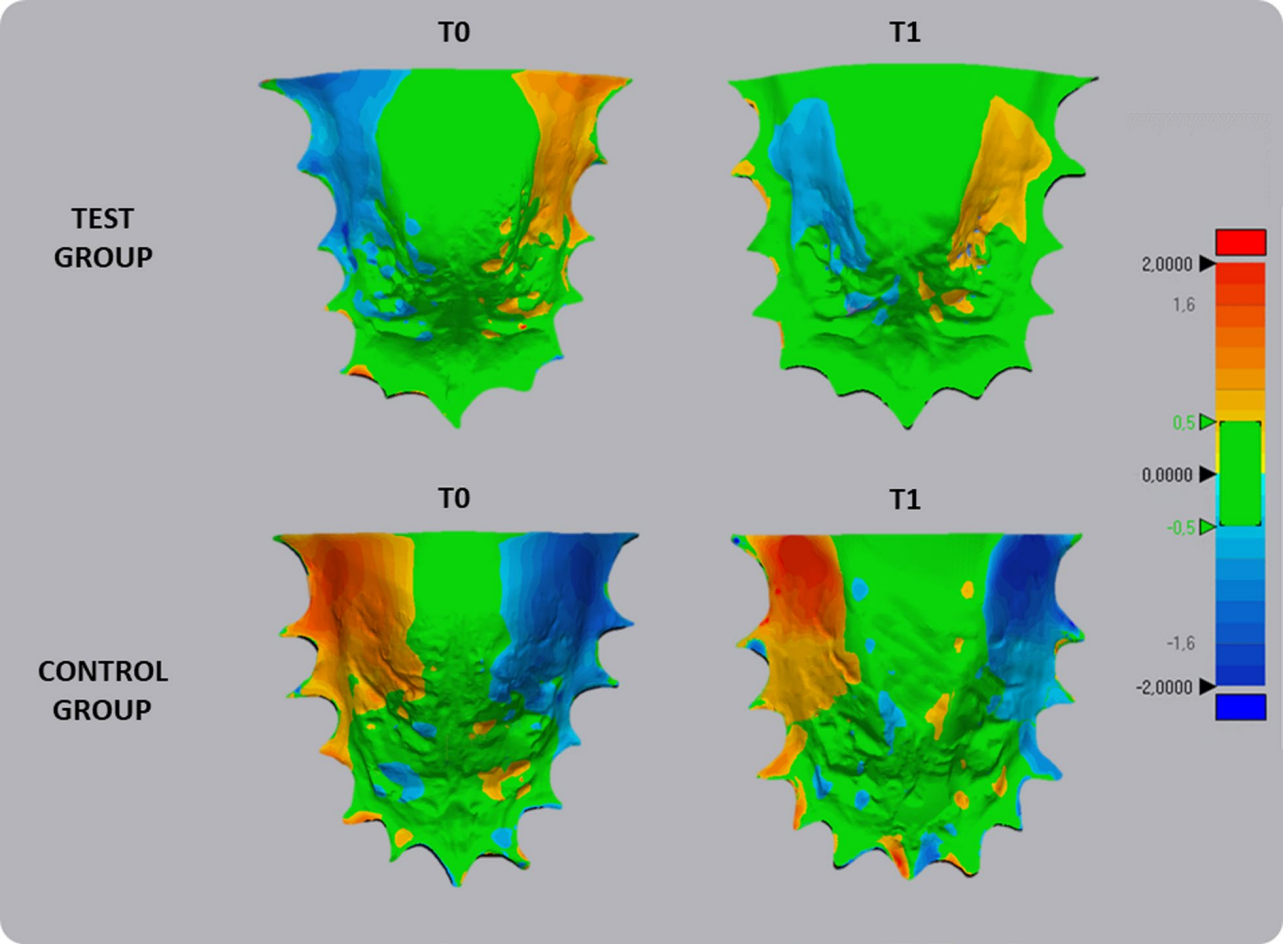
Deviation analysis and calculation of the percentage of matching between the original and mirrored models of the palate detected at T0 and T1 in both treated group (MEG) and control group (CG). The RGB colored scale bar (millimeters) is shown on the right: the upper (red) and lower (blue) parts of the scale indicate the maximum positive and negative deviations. Green indicates the tolerance range, set to 0.3 mm. The number of polygons for surface representation was set to the maximum of 100,000, and the corresponding polygons of the selected reference areas were automatically superimposed

**Fig. 4:**
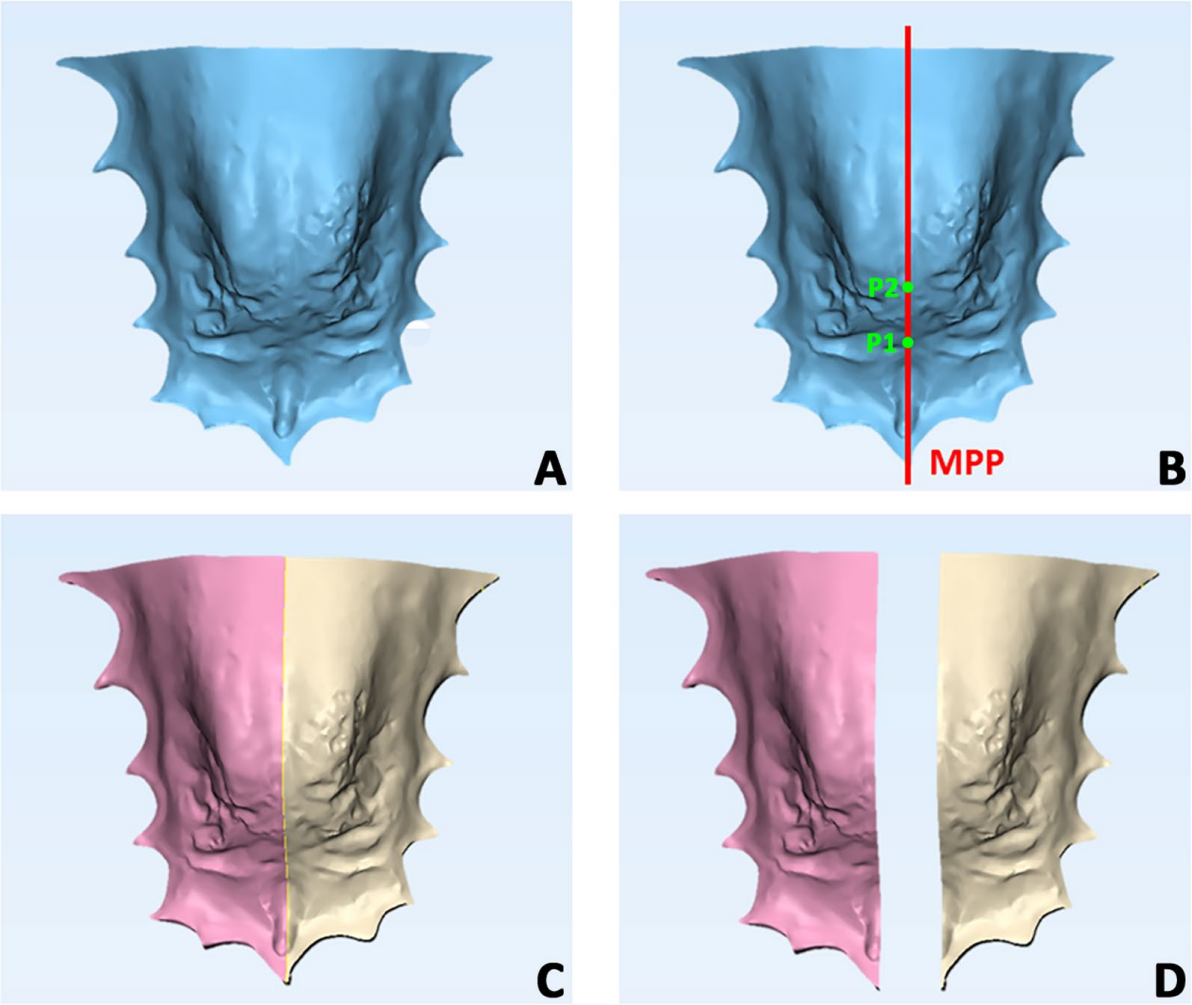
Generation of hemi-palatal volumes and volumetric assessment. **A**, **B** The same median palatal plane (MPP) drawn to perform the mirroring process was also used to split the maxillary model in to hemi-volumes. **C**, **D** Pink and golden models represent, respectively, the CBS and non-CBS volumes of the maxilla

To verify the morphological changes (symmetry/asymmetry) and perform surface analysis of the palate, a specific 3D imaging technology involving superimposition of T0 and T1 intra-oral scans was carried out, according to a consolidated methodology [4]. The procedure involved four steps: (1) Mirroring (3-matic Medical software, vr. 13, Materialise NV, Leuven, Belgium): the segmented palate was duplicated and mirrored using the midpalatal plane (MPP) as reference, that is the line passing through a point placed at the level of the second rugae and a second point 1 cm distal, along the palatal raphe (Fig. 2A–C). (2) Surface registration (3-Matic Medical software, vr. 13, Materialise NV, Leuven, Belgium): the original and mirrored palate models were superimposed via preliminary registration using MPP as reference plane, and a final registration was executed using the “Best-fit alignment” feature in the software (Fig. 2D). (3) Deviation analysis and matching percentage calculation (Geomagic Control™ X, version 2017.0.0, ‘3D Systems’, Rock Hill, USA): the mean and maximum values of the linear distances (Euclidean distance) between the surfaces of the two palatal models, measured across 100% of the surface points. The analysis was complemented by the visualization of the 3D color-coded maps, set at 0.3 mm range of tolerance (green color), to better evaluate and locate the discrepancy between the model surfaces (Fig. 3). These values represented the degree of correspondence between the original and the mirrored models and, therefore, provided quantitative data of the morphological characteristics of the palate detected at T0 and T1. (4) Volumetric assessment (3-Matic Medical software): total palate volume was calculated at T0 and T1 along with hemilateral volumes (CBS = cross-bite side, non-CBS = non-cross-bite side), the latter obtained using the same MPP used as reference for the mirroring procedure (Fig. 4A–D).

The entire workflow was carried out by a single expert operator (V.R.). After 4 weeks, the same measurements were repeated by the same operator to obtain data for intra-operator reliability. A second expert operator (A.L.G.) performed the digital workflow to obtain data for inter-operator reliability assessment.

### Study size

A pilot study was performed on 20 subjects satisfying the inclusion/exclusion criteria to evaluate sample size power. The analysis showed that 10 subjects for each group were required to detect mean difference of 204.47 mm^3^ between the intra-timing hemi-volumetric differences (cross-bite side and non-cross-bite side = asymmetry) recorded at T0 and T1, with a power of 80% and a significance level of 0.05. However, we were able to include 41 subjects increasing the robustness of the data findings.

### Statistics

Descriptive statistics was designed to assess the demographic and clinical characteristics of the study sample that could represent confounders influencing data outcomes. The numerical (age) and categorical (gender, skeletal maturity) characteristics between MEG and CG groups were compared by using the Student’s *t* test and Chi-square test that confirmed the balanced distribution of these factors (Table 1).

**Table 1:** Demography and clinical characteristics of the sample of the study

Sample characteristics	Total (*n* = 41)	MEG (*n* = 20)	CG (*n* = 21)	Significance
	Mean/*n*	Mean/*n*	Mean/*n*	
Mean age	7.8 (± 1.1)	8.1 (± 0.9)	7.7 (± 1.2)	0.267
Gender				
Male	17	9	8	0.895
Female	24	11	13	
Skeletal maturity				
CVMS 1	32	17	15	0.501
CVMS 2	9	3	6	

*p* value for comparison of group means by *t* test or differences in proportion calculated by chi-square test

Preliminary data analysis was performed using the Shapiro–Wilk test and Levene's test to assess data distribution and equality of variance. Since data showed normal data distribution, parametric tests were used. Unpaired Student’s *t* test was used to compare: (1) the mean difference of CBS/non-CBS between the two groups (inter-groups mean difference evaluation) at T0 and T1, (2) the mean difference of the T0-T1 surface’s matching of percentage between the two groups. Paired Student’s *t* test was used to assess: (1) hemilateral volumes between cross-bite side (CBS) and non-cross-bite side (non-CBS) (intra-timing assessment) at T0 and T1 in both groups, (2) inter-timing comparison of the mean differences of CBS/non-CBS between T0 and T1 in both group, (3) inter-timing comparison of the surface’s matching of percentage between T0 and T1 in both groups. Statistical significance was set at *p* < 0.05. Intra-examiner reliability was assessed using the Intraclass Correlation Coefficient (ICC). Datasets were analyzed using SPSS^®^ version 24 Statistics software (IBM Corporation, 1 New Orchard Road, Armonk, New York, USA).

## Results

### Main results

#### Volumetric measurements

A significant increment of the total palatal volume between T0 and T1 registrations was found in the MEG group and CG group (*p* < 0.05) and can be attributed, respectively, to the expansion of the maxilla (MEG group) and to normal growth (CG group) (Additional file 4: Table S2).

At T0, CBS was significantly smaller compared to the non-CBS in both groups (*p* < 0.05), suggesting an asymmetry of the maxillary arch. The inter-timing assessment revealed that in the MEG group the CBS /non-CBS mean differences was significantly reduced compared to T0 (*p* < 0.05). Accordingly, the inter-timing differences of the CBS/non-CBS differences were statistically significant between MEG and CG groups (*p* < 0.05) (Table 2).

**Table 2:** Comparative assessment of hemilateral palatal volumetric measurements recorded in the treated group (TG) and control group (CG)

Group	Timing	Side	Volume (mm^3^)	Diff	*p* value*	Mean diff. of the diff	*p* value**	*p* value***
MEG	T0	CBS	4817.94 (± 387.00)	334.46 (± 158.64)	< 0.0001	236.37 (± 134.33)	< 0.0001	< 0.0001
		Non-CBS	5152.40 (± 470.72)					
	T1	CBS	6028.00 (± 864.22)	98.09 (± 47.94)	0.736			
		Non-CBS	6126.09 (± 864.05)					
CG	T0	CBS	4877.56 (± 520.64)	408.36 (± 140.27)	< 0.0001	28.29 (± 72.02)	0.114	
		Non-CBS	5285.93 (± 463.60)					
	T1	CBS	5339.09 (± 582.89)	436.66 (± 124.57)	< 0.0001			
		Non-CBS	5775.75 (± 522.84)					

*CBS* Cross-bite side, *non-CBS* Non-cross-bite side, *NS* Non-significant

*p values based on paired Student’ *t* test for intra-timing side-to-side measurements and set at *p* < 0.05

**p values based on paired Student’ *t* test for inter-timing mean differences and set at *p* < 0.05

***p values based on Independent Student’ *t* test for inter-groups comparison of the mean of differences and set at *p* < 0.05

### Main results

#### Surface deviation analysis

At the baseline, the registration between the original and mirrored palatal models (T0/T0m) showed a limited percentage agreement in both groups, respectively, of 74.02% (± 9.83) in MEG and 76.36 (± 8.64) in the CG, suggesting a slight morphological asymmetry of the maxillary anatomy (Table 3). At T1, there was a statistically significant increment of the percentage of agreement of palatal surfaces (T1/T1m = 89.95% ± 7.12) in the MEG (*p* < 0.05). Accordingly, the mean difference between inter-timing recordings (T0/T0m vs T1/T1m) was statistically significant between the two groups (*p* < 0.05) (Table 3).

**Table 3:** Comparison of intra-timing matching percentage agreement between original and mirrored palate models in the study and control groups

		Matching %	*p* value*	Mean diff	*p* value**
MEG	T0/T0m	74.02 (± 9.83)	< 0.0001	15.93 (± 7.66)	< 0.0001
	T1/T1m	89.95 (± 7.12)			
CG	T0/T0m	76.36 (± 8.64)	0.199	4.18 (± 2.90)	
	T1/T1m	72.18 (± 9.5)			

T0/T0m = superimposition between original model and mirrored model at T0; T1/T1m = superimposition between original model and mirrored model at T1

*p value set at *p* < 0.05 based on paired Student’s *t* test for inter-timing comparisons

**p value based on Independent Student’s *t* test for inter-groups comparisons

Concerning deviation analysis, the palatal vault showed a prevalence of green color, indicating that this area coincided with the original and the mirrored models. Instead, the color-coded map showed an intense blue color on one side of the palatal surface of the alveolar process and an intense red color on the other. These data would suggest that the palatal asymmetry was mainly confined to the lower part of the palate at the alveolar processes level, as showed in (Fig. 4).

No differences were found between intra-operator readings, with excellent correlation indexes ranging from 0.911 to 0.935 for volumetric measurements and from 0.874 to 0.904 for surface analysis. Similarly, no differences were found between inter-operator readings, with excellent correlation indexes ranging from 0.896 to 0.924 for volumetric measurements and from 0.887 to 0.931 for surface analysis [11].

## Discussion

Most of the studies analyzing the asymmetry in subjects with FPXB had been focused on the mandible [12–16]. The available evidence suggests that FPXB determines an asymmetric position of the condyles in the glenoid fossa when compared to subjects with normal occlusion [12, 15]. As consequence, early treatment of FPXB is advocated to avoid the persistency of the condylar asymmetry during the growing stage which could interfere with mandibular function and bone remodeling [15–17].

On the contrary, few studies have been focused on the maxillary morphology and reported that FPXB in mixed dentition could be associated with asymmetric development of the maxillary arch [4, 18]. Such asymmetry may develop as adaptive and compensatory process aiming at invalidating functional deviation caused by mandibular shift toward the cross-bite side [19]. A recent study also suggested that maxillary asymmetry improves one year after treatment of FPXB with RME [7]. The knowledge of whether the observed baseline asymmetry is stable or can improve after treatment has significant clinical relevance when considering the future application of orthodontic biomechanics in permanent dentition or other dental rehabilitation procedures.

In the present study, we used a specific 3D imaging technology [4] that allowed the analysis of the symmetry of the palate vault to obtain a comprehensive evaluation of the morphological characteristics of the subjects affected by FPXB and the potential morphological changes that occurred due to treatment.

We included a control group of untreated subjects who postponed the treatment 12–24 months after the orthodontic consultation. The presence of a control group has permitted to discriminate the changes related to the treatment from those that occurred due to growth. Since subjects in the CG received the treatment between one or two years later, the present study does not introduce the ethical issues related to the recruitment of growing non-treated subjects for scientific purposes [20].

Findings at the baseline (T0) would confirm that subjects with FPXB (both MEG and CG groups) present an asymmetric morphology of the palate [18]. In general, the volumetric data detected at the cross-bite side (CBS) were significantly greater that those detected on the non-cross-bite side (non-CBS). These data reflected the limited percentage of agreement obtained by overlapping the original palate model with the mirrored model, with a surface correspondence of 74.02% in MEG and 89.95% in the CG. The color-coded map (Fig. 4) allows a qualitative evaluation of the surfaces’ discrepancy and showed that the mismatching between original and mirrored models was located in the palate region proximate to the dento-alveolar processes (blue-red colors), while surface data of the basal bone were within the range of tolerance. This pattern of mismatching would confirm that the asymmetry involved the alveolar process and has been explained as the adaptive bending of the maxillary alveolar process of the cross-bite side for maintaining occlusal contacts with the antagonist mandibular dentition due to mandibular shift [21]. The asymmetric functional pattern of mandibular muscles in FPXB growing patients could also contribute to this adaptive process [22]. A recent study conducted by Evangelista thoroughly examined the craniofacial asymmetry in children with transverse maxillary deficiency before and after RME. The authors of the study found that children with a narrow maxilla without FPXB exhibited greater asymmetries in the zygomatic arch and maxilla compared to subjects with FPXB. Although our study did not include subjects without FPXB, our findings corroborate those of the study of Evangelista, as both indicate an inherent adaptive or compensatory growth process in response to skeletal transverse deficiency. Consequently, it can be inferred that two distinct clinical conditions, namely the presence of FPXB or normal occlusal contact, represent two facets of the same phenomenon—an adaptive process that occurs during growth and that generate asymmetry in the presence of a transverse discrepancy between the maxillary and mandibular jaws. Longitudinal studies are necessary to assess the potential factors contributing to the occurrence or the absence of FPXB. From a clinical perspective, these findings emphasize the significance of carefully observing maxillary morphology, as it provides additional diagnostic insights that can be valuable in determining appropriate treatment approaches.

All subjects included in the MEG showed a complete correction of the FPXB at T1, with restore of the centric occlusion/centric relation ratio and midlines coincidence. The effectiveness of the treatment was confirmed by total palatal volumetric data that significantly increased after maxillary expansion. We found that the increment of the hemi-volume was significantly greater at the cross-bite side compared to the non-cross-bite side, reducing the mean difference between both sides detected at the baseline. The percentages of surfaces’ correspondence between original and mirrored models was 89.95%, and this value was significantly greater compared to the value recorded at the baseline. The color-coded map (Fig. 4) showed a similar pattern of asymmetry compared to the baseline; however, there was a remarkably extension of surface data agreement (tolerance = green color) toward the alveolar processes. Integrating the data obtained from volumetric and surfaces’ analysis, it is clear that subjects in the MEG showed a significant improvement in maxillary asymmetry after one year of therapy. As consequence, it could be assumed that the early correction of FPXB could contribute to restore the harmonious and more symmetric development of the palate, along with the correction of the malocclusion and of the associated mandibular dysfunction.

It is interesting to note that the maxillary expander screw works with a symmetric pattern of expansion, and that the expander used in the present study did not have asymmetric design of the framework (arms, resin pads, skeletal anchorage) that could have favored the expansion at the cross-bite side. Thus, one of the possible explanations for the improvement of the palatal asymmetry is that the correction of transverse discrepancy and the reestablishment of balanced occlusion may favor a spontaneous normal pattern of development of the palate and the alveolar processes.

Subjects included in this study had diagnosis of skeletal transverse maxillary deficiency and received slow maxillary expansion protocol (3 activations per week up to overcorrection) using Hyrax expander. The occurrence of skeletal expansion was clinically assumed by the appearance of the diastema between central incisors. In this regard, slow maxillary expansion generates similar dento-skeletal effect compared to RME, with less stress exerted on the midpalatal suture and less discomfort in children [23, 24]. However, since the study did not include radiographic examination of post-treatment changes, it was not possible to quantitative estimate either the amount of skeletal expansion on both sides or the ratio between skeletal and dento-alveolar effects. As consequence, it remains unclear if and how the skeletal effectiveness of the treatment (palatal bone expansion and bending of alveolar process) contributes to the main outcome of the present study, i.e. the improvement of the palatal asymmetry, or if the present findings can only be ascribed to the changes in the alveolar processes. With this notion in mind, it would be clinically worthy to investigate in future researches the quantitative and qualitative effects of different expansion protocol (rapid expansion vs slow expansion, skeletal expansion vs dento-alveolar expansion) and different treatment timing (different skeletal maturation stages) in the post-treatment evolution of the maxillary asymmetry, using radiographic acquisitions.

All the subjects in the CG group reported FPXB at T1, confirming previous evidence that FPXB is not self-correcting and requires early treatment to prevent the malocclusion from eventually being perpetuated in permanent dentition [25]. In the CG, the total palatal volume was slightly greater at T1 compared to T0, suggesting the occurrence of growing process at developmental stage (Additional file 4: Table S2). However, data from hemi-volumes assessment and surface analysis (matching percentage and color-code map) showed a slight worsening of the asymmetry detected at the baseline, although without statistical significance (Tables 2 and 3).

The maintenance of the asymmetry in the CG could be explained by the persistence of the FPXB one year later and of those factors influencing the adaptive processes above-mentioned.

In this regard, Primozic et al. [26] reported that the mixed dentition phase is the critical stage for the development of facial asymmetry in subjects affected by FPXB, and that this malocclusion should be intercepted at this stage rather than in deciduous dentition (asymmetry not expressed) or permanent dentition (consolidated bony asymmetry). Consistently with this assumption, our findings would suggest that the choice for treatment of FPXB in mixed dentition should be based on the aim to restore form and function [27]. Also, from the orthodontic clinical perspective, the possibility to obtain the spontaneous enhancement of palatal symmetry would reduce the severity of dento-alveolar compensations that complicate the biomechanics of expansion at a later stage.

### Limitations

- The small sample size and the limited control group are the major concerns of the present investigation. However, considering the general ethical restriction related to the recruitment of untreated subjects for scientific purposes, the present control group represents a heritage sample that could be used for further comparative investigations.
- Another important limitation of the study is that the observation time is limited to a maximum of 18 months after treatment. Future studies could investigate the maintenance of post-treatment changes in the long term; however, this would entail extending the observational time even in the control group, with inherent ethical issues or difficulties in retrieving non-treated subjects.
- Both treatment and control groups were retrospectively recruited. Thus, it was impossible to control a priori potential confounding factors or specific variables that may have affected data outcomes. However, according to the inclusion/exclusion criteria, we were able to analyze a homogeneous study sample concerning mean age, dental and skeletal maturation stage. Future randomized studies are warmly encouraged to overcome the reported limitation of study design.

## Conclusions

Although the primary goal of treating FPXB is the correction of the malocclusion and of the associated altered mandibular function, the present findings would suggest that the early treatment of this condition could favor a more harmonious and symmetric development of the palate.

### Supplementary Information

**Additional file 1: Figure S1.** Example of palatal expander used in the MEG group. **A** Appliance in place before activation protocol. **B** Appliance in place after expansion.

**Additional file 2: Figure S2.** STROBE flow chart showing the included study sample retrieved from a retrospective cohort of subjects with diagnosis of transverse maxillary deficiency. STROBE, Strengthening the Reporting of Observational Studies in Epidemiology.

**Additional file 3: Table S1.** STROBE Statement—checklist of items that should be included in reports of observational studies.

**Additional file 4: Table S2.** Total palatal volume changes recorded in the treated group (MEG) and control group (CG).

## Data Availability

The datasets used and/or analyzed during the current study are available from the corresponding author on reasonable request.

## Abbreviations

FPXB: Functional posterior cross-bite
RMS: Root mean square
RME: Rapid maxillary expansion
MEG: Maxillary expansion group
CG: Control group
MPP: Midpalatal plane
CBS: Cross-bite side
Non-CBS: Non-cross-bite side
