# Supplementary figures and images for: Analysis of maxillary asymmetry before and after treatment of functional posterior cross-bite: a retrospective study using 3D imaging system and deviation analysis
Source: Prog Orthod. 2023 Dec 11;24:41. doi: 10.1186/s40510-023-00494-z (PMC10710971; doi:10.1186/s40510-023-00494-z)

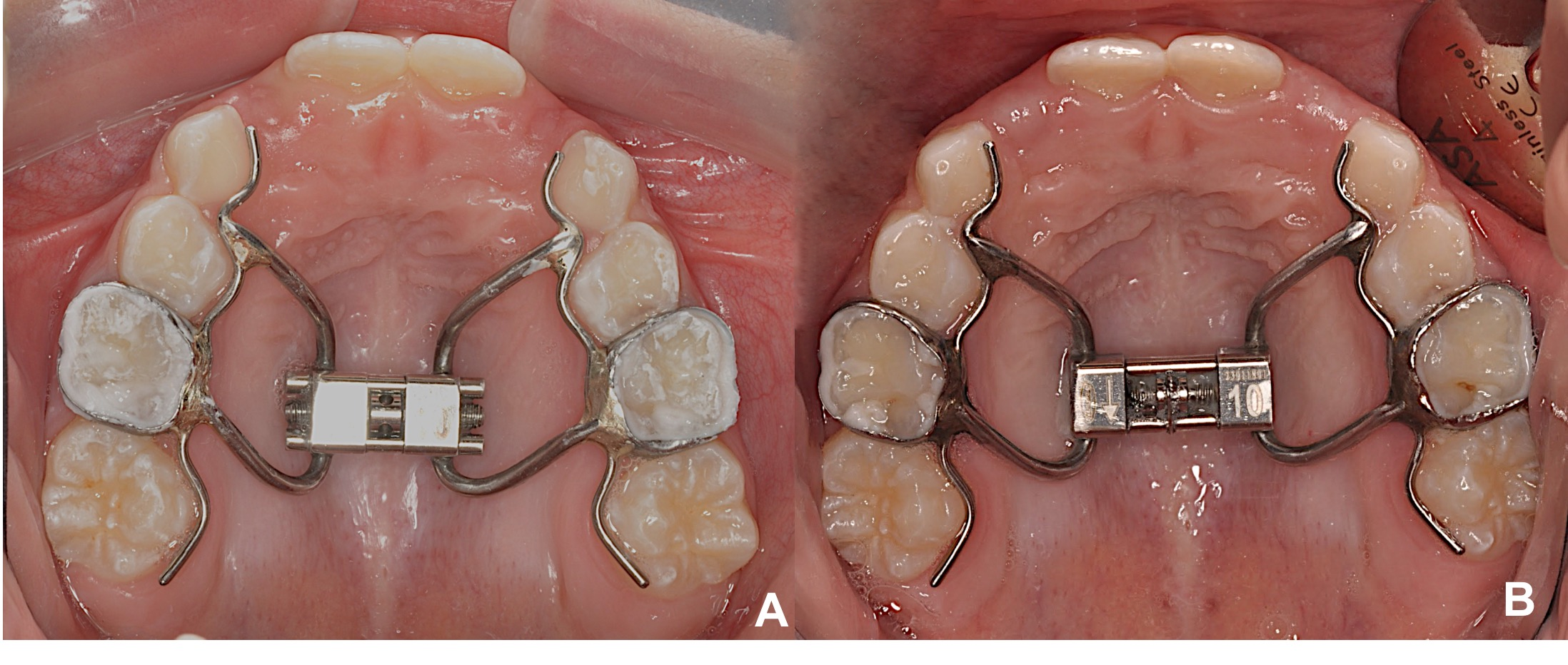

Supplement: Supplementary file 1 — Additional file 1: Figure S1. Example of palatal expander used in the MEG group. A Appliance in place before activation protocol. B Appliance in place after expansion. [file 40510_2023_494_MOESM1_ESM.tiff]

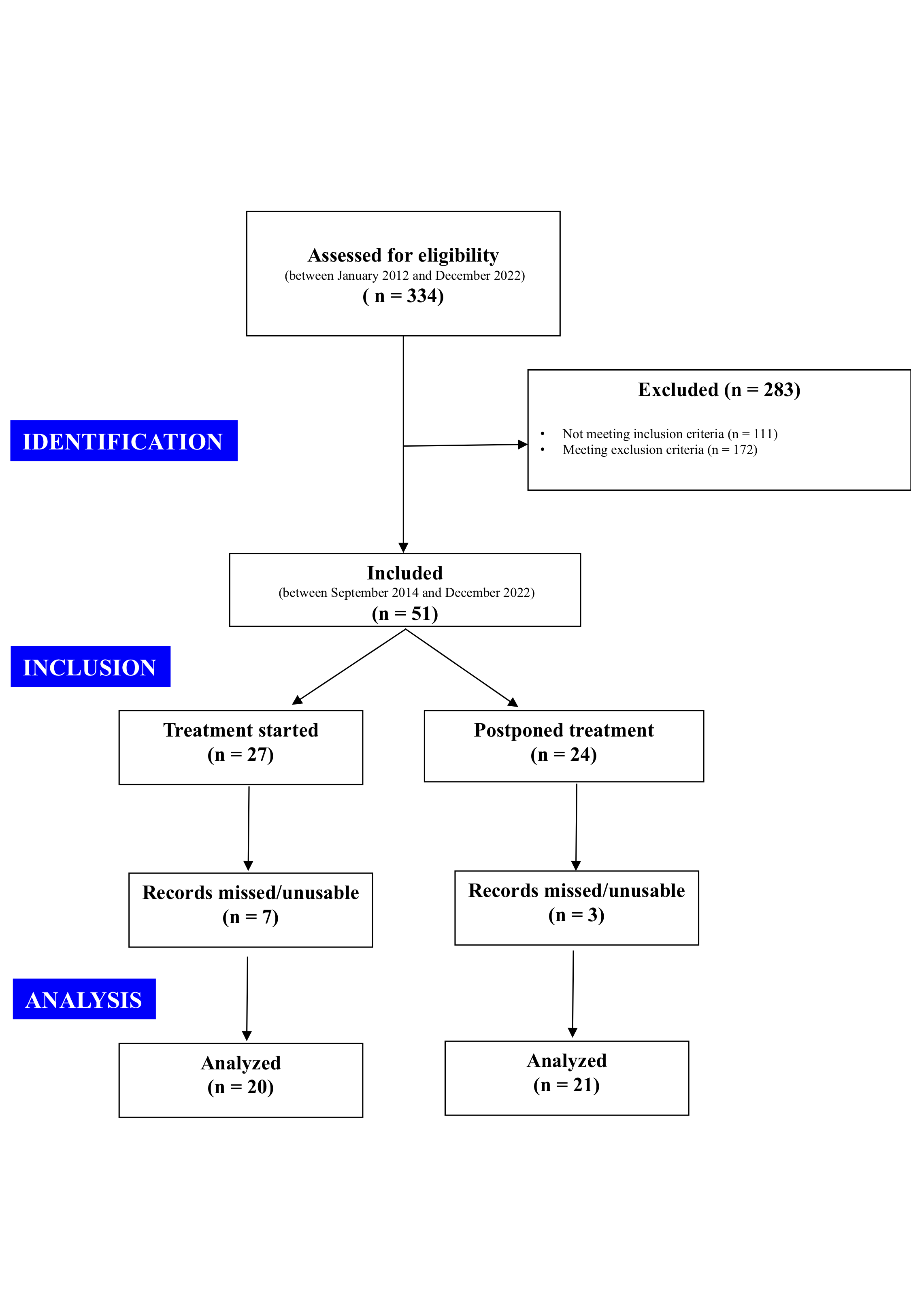

Supplement: Supplementary file 2 — Additional file 2: Figure S2. STROBE flow chart showing the included study sample retrieved from a retrospective cohort of subjects with diagnosis of transverse maxillary deficiency. STROBE, Strengthening the Reporting of Observational Studies in Epidemiology. [file 40510_2023_494_MOESM2_ESM.tiff]
