# Supplementary material for: Analysis of maxillary asymmetry before and after treatment of functional posterior cross-bite: a retrospective study using 3D imaging system and deviation analysis
Source: Prog Orthod. 2023 Dec 11;24:41. doi: 10.1186/s40510-023-00494-z (PMC10710971; doi:10.1186/s40510-023-00494-z)
Supplement: Supplementary file 3 — Additional file 3: Table S1. STROBE Statement—checklist of items that should be included in reports of observational studies. [file 40510_2023_494_MOESM3_ESM.docx]

|  | Item No. | Recommendation | Page  No. | Relevant text from manuscript |
| --- | --- | --- | --- | --- |
| **Title and abstract** | 1 | (*a*) Indicate the study’s design with a commonly used term in the title or the abstract | 1 | - |
|  |  | (*b*) Provide in the abstract an informative and balanced summary of what was done and what was found | 1-2 | - |
| Introduction | | | |  |
| Background/rationale | 2 | Explain the scientific background and rationale for the investigation being reported | 2-3 | - |
| Objectives | 3 | State specific objectives, including any prespecified hypotheses | 3-4 | - |
| Methods | | | |  |
| Study design | 4 | Present key elements of study design early in the paper | 4 | - |
| Setting | 5 | Describe the setting, locations, and relevant dates, including periods of recruitment, exposure, follow-up, and data collection | 4-5 | - |
| Participants | 6 | *Case-control study*—Give the eligibility criteria, and the sources and methods of case ascertainment and control selection. Give the rationale for the choice of cases and controls | 5 | - |
| Variables | 7 | Clearly define all outcomes, exposures, predictors, potential confounders, and effect modifiers. Give diagnostic criteria, if applicable | 5 | - |
| Data sources/ measurement | 8 | For each variable of interest, give sources of data and details of methods of assessment (measurement). Describe comparability of assessment methods if there is more than one group | 6-7 | *-* |
| Bias | 9 | Describe any efforts to address potential sources of bias | 7 | - |
| Study size | 10 | Explain how the study size was arrived at | 7 | - |

Continued on next page

| Quantitative variables | 11 | Explain how quantitative variables were handled in the analyses. If applicable, describe which groupings were chosen and why | 7 | - |  |
| --- | --- | --- | --- | --- | --- |
| Statistical methods | 12 | (*a*) Describe all statistical methods, including those used to control for confounding | 7-8 | - |  |
|  |  | (*b*) Describe any methods used to examine subgroups and interactions | 7-8 | - |  |
|  |  | (*c*) Explain how missing data were addressed | - | - |  |
|  |  | *(d) Case-control study*—If applicable, explain how matching of cases and controls was addressed | 7, Table 1 | - |  |
|  |  | (*e*) Describe any sensitivity analyses | - | - |  |
| Results | | | | |  |
| Participants | 13 | (a) Report numbers of individuals at each stage of study—eg numbers potentially eligible, examined for eligibility, confirmed eligible, included in the study, completing follow-up, and analysed | 8, Supplementary Figure 1 | - |  |
|  |  | (b) Give reasons for non-participation at each stage | Supplementary Figure 1 |  |  |
|  |  | (c) Consider use of a flow diagram | Supplementary Figure 1 |  |  |
| Descriptive data | 14 | (a) Give characteristics of study participants (eg demographic, clinical, social) and information on exposures and potential confounders | Table 1 | - |  |
|  |  | (b) Indicate number of participants with missing data for each variable of interest | - | - |  |
| Outcome data | 15 | *Case-control study—*Report numbers in each exposure category, or summary measures of exposure | 8-9 | | |
| Main results | 16 | (*a*) Give unadjusted estimates and, if applicable, confounder-adjusted estimates and their precision (eg, 95% confidence interval). Make clear which confounders were adjusted for and why they were included | - | - |  |
|  |  | (*b*) Report category boundaries when continuous variables were categorized | - | - |  |
|  |  | (c) If relevant, consider translating estimates of relative risk into absolute risk for a meaningful time period | - | - |  |

| Other analyses | 17 | Report other analyses done—eg analyses of subgroups and interactions, and sensitivity analyses | - | - |
| --- | --- | --- | --- | --- |
| Discussion | | | | |
| Key results | 18 | Summarise key results with reference to study objectives | 10-14 | - |
| Limitations | 19 | Discuss limitations of the study, taking into account sources of potential bias or imprecision. Discuss both direction and magnitude of any potential bias | 14 |  |
| Interpretation | 20 | Give a cautious overall interpretation of results considering objectives, limitations, multiplicity of analyses, results from similar studies, and other relevant evidence | 10-14 |  |
| Generalisability | 21 | Discuss the generalisability (external validity) of the study results | 10-11 |  |
| Other information | |  | | |
| Funding | 22 | Give the source of funding and the role of the funders for the present study and, if applicable, for the original study on which the present article is based | - |  |

**Supplementary Table 1.** STROBE Statement—checklist of items that should be included in reports of observational studies
