# Supplementary material for: Analysis of maxillary asymmetry before and after treatment of functional posterior cross-bite: a retrospective study using 3D imaging system and deviation analysis
Source: Prog Orthod. 2023 Dec 11;24:41. doi: 10.1186/s40510-023-00494-z (PMC10710971; doi:10.1186/s40510-023-00494-z)
Supplement: Supplementary file 4 — Additional file 4: Table S2. Total palatal volume changes recorded in the treated group (MEG) and control group (CG). [file 40510_2023_494_MOESM4_ESM.docx]

|  | **Volume (mm3)** | | | | | |  | |  | |  | |  | |  | |  | |  | |
| --- | --- | --- | --- | --- | --- | --- | --- | --- | --- | --- | --- | --- | --- | --- | --- | --- | --- | --- | --- | --- |
|  | **T0** | **T1** | **p value*** | **T1-T0** | **p value**** |  | |  | |  | |  | |  | |  | |  | |  |
| **MEG** | 9970,34 (±847,07) | 12154,09 (±1727,61) | **p < 0,0001** | 2183,76 (±1349,08) | **p < 0,0001** |  | |  | |  | |  | |  | |  | |  | |  |
|  |  |  |  |  |  |  | |  | |  | |  | |  | |  | |  | |  |
| **CG** | 10163,49 (±975,87) | 11114,84 (±1100,33) | **p < 0,0001** | 951,35 (±285,40) |  |  | |  | |  | |  | |  | |  | |  | |  |
|  |  |  |  |  |  |  | |  | |  | |  | |  | |  | |  | |  |
|  |  |  |  |  |  |  | |  | |  | |  | |  | |  | |  | |  |
| **Supplementary Table 2**. Total palatal volume changes recorded in the treated group (MEG) and control group (CG) | | | | | | | | | | | | | | | | | | | | |
| *P values based on paired Student' t test and set at p < 0.05 (inter-timing comparison); **P values based on Independent Student' t test and set at p < 0.05 (inter-groups comparison) | | | | | | | | | | | | | | | | | | | | |
